# Are There Any Significant Risk Factors Associated with Lateral Trochanteric Pain in Patients Who Have Undergone Primary Hip Replacement?

**DOI:** 10.7759/cureus.44863

**Published:** 2023-09-07

**Authors:** Hilmi Alkan, Yavuz Karaman, Şahan Güven, Vedat Biçici, İzzet Özay Subaşı, Niyazi Erdem Yaşar, Ahmet Fırat

**Affiliations:** 1 Orthopaedics and Traumatology, Ankara Bilkent City Hospital, Ankara, TUR; 2 Orthopaedics and Traumatology, Erzincan University Faculty of Medicine, Erzincan, TUR

**Keywords:** pain of hip, trochanteric bursa repair, subcutaneous fat thickness, lateral trochanteric pain, total hip arthroplasty (tha)

## Abstract

Introduction

Lateral pain around the greater trochanter (LTP) is a common complication after total hip arthroplasty (THA) that can significantly reduce quality of life. The aim of this study was to analyze the relationship between lateral trochanteric bursa repair, subcutaneous fat thickness, and trochanteric pain during the THA procedure.

Materials and methods

A total of 98 patients who underwent THA for hip arthrosis between 2021 and 2022 were evaluated retrospectively. For all evaluated patients, subcutaneous thickness was measured between the fascia and the skin at the incision site. Bursa repair was performed in 47 patients, while bursa excision was done in 51 patients. The data obtained included demographic information, functional scores, comorbidities, bursa repair and skin thickness values, radiographic evaluations, and other specific markers. These were compared between patients diagnosed with LTP following THA and the controls.

Results

No difference was observed between the study groups in terms of subcutaneous fat thickness, bursa repair, and other demographic or radiographic evaluations. As expected, there were statistically significant differences between the groups in terms of the visual analog scale (VAS) score (p=0.030) and the Harris hip score (HHS) (p=0.045). When comparing the groups with and without LTP, the VAS score was higher in the group with LTP, while the HHS was found to be lower.

Conclusion

Trochanteric pain is not associated with bursa repair or subcutaneous thickness. LTP cannot be predicted based on comorbidities such as smoking, BMI, or radiographic measurements.

## Introduction

The relationship between obesity and the risk of complications following total hip arthroplasty (THA) has been the subject of comprehensive research [1]. In the presence of excessive fat at the incision site, there is a potential increase in dead space, wound tension, and fat necrosis, which may subsequently lead to an indirect reduction in blood supply to the wound area, thereby contributing to surgical site complications [2]. Numerous studies have thus far measured subcutaneous thickness on X-ray and CT images post-THA to explore their association with post-surgical complications [3]. However, there is a paucity of research examining the relationship between clinically measured incision site fat thickness during the surgical procedure and its impact on THA outcomes, particularly concerning its association with lateral trochanteric pain (LTP) [3]. In this context, understanding the distribution of subcutaneous fat around the hip region has the potential to offer deeper insights into the pathophysiological mechanisms underlying obesity and its implications in THA complications. Such investigations can contribute to a better understanding of how obesity influences the healing process and post-surgical outcomes following THA. Consequently, these findings may have practical implications for optimizing surgical approaches and minimizing complications for patients undergoing THA, especially those with obesity. The occurrence of LTP following THA can present significant challenges for patients, affecting their daily activities and overall quality of life. The etiology of LTP seems to be multifactorial, with various surgical parameters, patient-related factors, and biomechanical changes post-THA being explored for their association with pain development around the trochanteric area. However, a clear consensus remains elusive. The lack of definitive evidence has led to differing opinions among surgeons regarding the optimal intraoperative management of the trochanteric bursa. One proposed approach is bursa repair, and the other is resection. During THA using the posterolateral approach, the trochanteric bursa is often cut or resected before the external rotators are severed at the adhesion site. Some surgeons prefer complete excision of the bursa, believing it may be a contributing factor to postoperative LTP [4]. Conversely, others argue for the preservation of bursal tissue lateral to the large trochanter, creating a barrier over the trochanter and posterior region to reduce pain by minimizing friction between soft tissues and bone [4]. The primary objectives of this study were twofold: (1) to assess the occurrence of LTP following THA by comparing its incidence between patients who underwent bursa repair and those who did not, and (2) to investigate the associations between trochanteric pain and various factors, including subcutaneous fat thickness, demographic characteristics, comorbidities, as well as radiological and clinical changes.

## Materials and methods

This study received approval from the Local Ethics Committee Board. A single senior physician, Ahmet Fırat, conducted all clinical and radiological data measurements. The retrospective evaluation included a total of 98 patients who underwent THA using a posterolateral approach due to hip osteoarthritis. They were followed up for a minimum of 1 year between 2021 and 2022. The study applied specific exclusion criteria. These criteria excluded patients who had undergone THA with a lateral offset femoral stem, individuals aged below 40 or above 75 years, those with a history of previous surgical interventions on the ipsilateral hip and thigh, documented tendinopathy or microtrauma of the gluteus medius and minimus tendons, prior corticosteroid injections into the trochanteric region within the year before THA, significant defects in the hip joint bone, leg length discrepancies exceeding 2.5 cm, chronic low back pain, malignant diseases, inflammatory arthritis, metabolic diseases, and severe musculoskeletal or bone disorders. Demographic variables, smoking status, and the side on which the surgery was performed were recorded. Surgical data were documented, including bursa repair and subcutaneous fat thickness measurements. Before the surgery, patients underwent both clinical and radiographic evaluations, which involved a detailed medical history, physical examination, and radiographs of the pelvis and hip (including anterior-posterior (AP) pelvis, AP hip, and lateral hip), conducted by a different nonoperative physician. Patients were scheduled for regular follow-up visits at the medical office after surgery. The diagnosis of LTB after THA was made based on the following criteria: (1) patients reported hip flank pain localized to the trochanteric area in a sideways position; (2) perceptible tenderness and/or pain on the large trochanter when subjected to the FABER test in supine lying position; and (3) the absence of any other apparent causes that could explain the patient's symptoms and complaints, such as postoperative infection, fracture, dislocation, or the need for revision surgery of the operated hip [5, 6].

During the surgical procedure, routine intraoperative fat measurements were performed at the incision site immediately after making the first incision. A standard, sterile ruler was introduced into the wound at the central part of the surgical area, perpendicular to the incision, after the incision had been opened between the skin and the fascia lata. The distance between the ruler and the iliotibial band and the skin was measured [1]. In the group of patients who underwent bursa repair, the external rotators were sutured instead of being left adhered, and they were subsequently closed using bursa no. 1 absorbable continuous suture (Vicryl Ethicon). Conversely, in the group that did not undergo bursa repair, only the external rotators were sutured at the adhesion site, and the bursa tissue was excised [4]. Standardized preoperative and postoperative AP pelvic X-rays were used for radiographic measurements. The measurements were conducted on the operated hip, while the non-surgical hip served as a control. Four different measurements were obtained as follows: (1) femoral offset: This measurement involved calculating the distance from the center of rotation of the femoral head to a line that intersects the long axis of the femur; (2) lateral large trochanter width: It referred to the ratio of the horizontal length from the midline to the anterior inferior iliac spine to the horizontal length from the midline to the lateral alignment of the large trochanter; (3) leg length discrepancy (LLD): LLD was determined by measuring the perpendicular distances from the bi-ischial line to the tip of each of the smaller trochanters, taking the femoral reference; (4) lateralization: This measurement involved calculating the distance from the medial teardrop to the center of the femoral head; and (5) vertical position: The steep distance from the middle of the femoral head to the teardrop was measured. The radiographic evaluation was performed by an author who was not involved in the surgical procedures of this study [7, 8]. The visual analog scale (VAS) and Harris hip score (HHS) values were recorded immediately before the surgery and during the final follow-up assessments.

Statistical analysis

Descriptive data are summarized as either frequency (percentages) or median (interquartile ranges (IQR): 25th-75th percentiles), depending on the data distribution. The Shapiro-Wilk test was used to assess the normality of the continuous variables. Due to non-normality, the Mann-Whitney U test was used to compare continuous data between groups. Differences in preoperative and postoperative measurements were assessed using the Wilcoxon signed-rank test. Pearson's chi-square test was used to evaluate the associations between categorical variables. Analyses were performed using the SPSS v26.0 for Windows (IBM Corp., Armonk, NY, USA). A p-value of <0.05 was considered statistically significant.

## Results

A total of 98 individuals (59 females, 39 males) were included in the study. The median age of participants was 56 years (IQR: 44-65). The comparison of characteristics between patients with and without pain at the one-year follow-up is presented in Table 1. There were no significant differences in demographic data between the study groups. As expected, statistically significant differences were observed between the groups in terms of the VAS score (p=0.030) and the HSS score (p=0.045). When comparing the group with LTP to the group without LTP, the VAS score was higher in the LTP group, while the HSS score was lower.

**Table 1:** Comparison of patient characteristics between patients with and without pain. Values are expressed as medians (25th, 75th percentiles) or frequencies (percentages). For categorical variables, Pearson’s chi-square tests were used. Continuous variables were compared using the Mann-Whitney U tests. Bold p-values indicate statistically significant differences between groups.

Variables	No Pain (n=64)	Pain (n=34)	P-value
Age, years	58.0 (45.0, 65.0)	54.5 (43.3, 61.3)	0.169
Gender			0.507
Male	27 (42.2%)	12 (35.3%)	
Female	37 (57.8%)	22 (64.7%)	
BMI, kg/m2	29.9 (26.4, 33.3)	30.4 (26.5, 33.0)	0.547
Smoking, yes	27 (42.2%)	8 (23.5%)	0.067
Side			0.794
Right	34 (53.1%)	19 (55.9%)	
Left	30 (46.9%)	15 (44.1%)	
Bursa repaired	27 (42.2%)	20 (58.8%)	0.117
Subcutaneous fat thickness, mm	35.0 (24.0, 49.3)	33 (28.0, 56.5)	0.545
VAS score	0 (0, 1)	1 (0, 2)	0.030
HSS score	90.5 (86.0, 93.0)	85.8 (78.4, 93.5)	0.045

In Table 2, the changes in patient data from pre-op to post-op measurements were evaluated. No statistically significant differences were found between patients with and without pain regarding the change scores. Although not significant, the most substantial difference in change scores between the groups was observed in horizontal position lateralization. In the patients with pain, the decrease in horizontal position lateralization from pre-op to post-op was smaller (median: -4.5, IQR: -10.1 to -1.1) compared to those without pain (median: -9.5, IQR: -14.8 to -0.5).

**Table 2:** Comparison of change in patient data from pre-op to post-op between patients with and without pain. Values are expressed as median (25th, 75th percentiles). P-values were calculated using Mann-Whitney U tests.

Change score (Postop-preop)	No Pain (n=64)	Pain (n=34)	P-value
Femoral offset	4.8 (-1.5, 10.6)	5.6 (-1.6, 7.0)	0.296
Lateral GT Width	-0.01 (-0.06, 0.04)	0.01 (-0.02, 0.05)	0.108
Leg Length	6.5 (-0.3, 23.0)	4.0 (-0.5, 12.0)	0.125
Horizontal Position Lateralisation	-9.5 (-14.8, -0.5)	-4.5 (-10.1, -1.1)	0.086
Vertical Position	-1.7 (-5.7, 1.1)	0.4 (-5.0, 2.2)	0.305

Table 3 compares the differences between pre-op and post-op measurements for patients with and without pain. For both patient groups, the differences between pre-op and post-op measurements of femoral offset (p (no pain group) < 0.001, p (pain group) = 0.004), leg length (p (no pain group) < 0.001, p (pain group) = 0.015), and horizontal position lateralization (p (no pain group) < 0.001, p (pain group) < 0.001) were statistically significant. Following surgery, femoral offset and leg length increased, and horizontal position lateralization decreased in both groups. The decrease in vertical position was found to be significant in the patients without pain (p=0.002), while it was not found to be significant in the patients with pain (p=0.241).

**Table 3:** The comparison of preoperative and postoperative measurements across patients with and without pain. Values are expressed as median (25th, 75th percentiles). P-values were calculated with Wilcoxon signed rank tests. Bold p-values indicate statistically significant difference between the preop and postop measurements.

	No Pain (n=64)			Pain (n=34)		
	Pre-op	Post-op	p-value	Pre-op	Post-op	p-value
Femoral offset	36.8 (29.7, 42.7)	41.0 (37.2, 46.8)	<0.001	32.8 (30.0, 42.1)	36.5 (31.5, 44.8)	0.004
Lateral GT Width	0.84 (0.74, 0.88)	0.79 (0.75, 0.84)	0.257	0.82 (0.74, 0.93)	0.81 (0.76, 0.91)	0.152
Leg Length	-6.0 (-11.0, -0.8)	0.0 (-6.0, 9.9)	<0.001	-3.0 (-7.5, 0.0)	0.0 (-1.3, 8.0)	0.015
Horizontal Position Lateralisation	39.0 (36.0, 47.0)	33.2 (29.5, 34.6)	<0.001	37.0 (34.0, 43.3)	34.8 (28.3, 35.6)	<0.001
Vertical Position	23.0 (19.8, 27.9)	21.4 (17.9, 27.6)	0.002	22.0 (15.0, 24.3)	18.8 (17.0, 23.6)	0.241

## Discussion

The key findings of this study indicate that neither bursa repair nor subcutaneous fat thickness is associated with the development of LTP following THA. These results can be attributed to the fact that while the trochanteric bursa acts as a cushion over structures situated more posteriorly, such as the external rotators, it does not fully cover structures located laterally, such as the large trochanter. Additionally, even if the bursa covers the lateral structures partially, the repaired portion cannot be fully restored to its original position. As for the relationship with subcutaneous fat thickness, the results can be attributed to trochanteric pain being primarily influenced by friction between the tensor fascia lata and the trochanter major. The thickness of the subcutaneous tissue does not significantly impact this distance or friction between the mentioned structures. Following THA, LTP is a recognized phenomenon, with reported prevalence ranging from 4% to 17% [9,10]. The etiology of LTP has primarily been attributed to scar tissue formation after surgery and the development of trochanteric bursitis [4]. However, histological examinations of bursa tissues from patients who underwent THA and were diagnosed with LTP revealed no evidence of acute or chronic inflammation, leading to the inability to confirm this theory [11,12]. Therefore, it is not entirely accurate to equate the diagnosis of LTP with bursitis and solely attribute the root cause of the problem to it. Chalidis BE et al. compared patients who underwent posterolateral intervention and primary THA and those who received bursa repair and resection [4]. They found an increase in hip movements during the early postoperative period and less pain when patients lay on the side of the surgery in the bursa repair group. In our study, we evaluated patients who were followed up for at least one year, but no significant difference in pain was observed between the groups. We believe that this discrepancy may be attributed to the duration of postoperative follow-up.

The association between alterations in hip biomechanics and the development of LTP after THA has been a subject of ongoing inquiry. However, despite acquiring diverse data related to implant design and placement, no definitive conclusions have been reached due to conflicting results [13]. One hypothesis suggests that excessive femoral protrusion, attempted to enhance THA stability, may lead to the prominence of the large trochanter, thereby causing LTP. Additionally, certain studies have indicated that leg extension can induce changes in gait patterns and force distribution around the hips by tightening soft tissues [14]. Conversely, other studies have reported no correlation between changes in leg length, lateralization of the femoral head center, and the incidence of LTP after THA [4,8]. Upon reviewing these studies, it becomes evident that many of them solely drew conclusions based on postoperative data without comparing preoperative and postoperative radiological values of the groups with and without pain. For instance, Farmer KW et al. examined patients diagnosed with LTP after primary THA who received cortisone injections. They observed differences in leg length in most of these patients, but definitive results could not be obtained as they did not compare them with a group that did not experience pain [15]. Our study has several limitations that should be acknowledged. Firstly, the number of patients included in the study was relatively small. A larger sample size could have potentially yielded more statistically significant results, particularly when analyzing risk factors. Secondly, our study design was retrospective, which may introduce inherent biases and limitations associated with such an approach. Lastly, although all patients received a standard offset type of prosthesis, two different brands of prostheses were used due to changes in the payment scope by the state. This difference in prosthesis brands could potentially introduce confounding variables and influence the study's outcomes.

## Conclusions

In conclusion, while the specific factors contributing to the development of LTP after THA are not fully understood, our study suggests that neither trochanteric bursa repair nor resection, nor subcutaneous thickness during posterolateral hip approaches, is associated with the occurrence of LTP. Additionally, LTP does not appear to be predictable based on factors such as comorbidities (e.g., smoking, body mass index) or radiographic measurements.
